# The Corn Smut (‘Huitlacoche’) as a New Platform for Oral Vaccines

**DOI:** 10.1371/journal.pone.0133535

**Published:** 2015-07-24

**Authors:** Margarita Juárez-Montiel, Andrea Romero-Maldonado, Elizabeth Monreal-Escalante, Alicia Becerra-Flora, Schuyler S. Korban, Sergio Rosales-Mendoza, Juan Francisco Jiménez-Bremont

**Affiliations:** 1 Laboratorio de Estudios Moleculares de Respuesta a Estrés en Plantas, División de Biología Molecular, Instituto Potosino de Investigación Científica y Tecnológica AC, San Luis Potosí, San Luis Potosí, México; 2 Laboratorio de Biofarmacéuticos Recombinantes, Facultad de Ciencias Químicas, Universidad Autónoma de San Luis Potosí, San Luis Potosí, San Luis Potosí, México; 3 Department of Biology, University of Massachusetts Boston, Boston, Massachusetts, United States of America; University of Massachusetts Medical School, UNITED STATES

## Abstract

The development of new alternative platforms for subunit vaccine production is a priority in the biomedical field. In this study, *Ustilago maydis*, the causal agent of common corn smut or ‘huitlacoche’has been genetically engineered to assess expression and immunogenicity of the B subunit of the cholera toxin (CTB), a relevant immunomodulatory agent in vaccinology. An oligomeric CTB recombinant protein was expressed in corn smut galls at levels of up to 1.3 mg g-1 dry weight (0.8% of the total soluble protein). Mice orally immunized with ‘huitlacoche’-derived CTB showed significant humoral responses that were well-correlated with protection against challenge with the cholera toxin (CT). These findings demonstrate the feasibility of using edible corn smut as a safe, effective, and low-cost platform for production and delivery of a subunit oral vaccine. The implications of this platform in the area of molecular pharming are discussed.

## Introduction

Following infection of young maize seedlings with the fungus *Ustilago maydis*, tumors or galls are formed in all aerial parts of these plants, and these galls are known as corn smut or ‘huitlacoche’[1–2]. Interestingly, ‘huitlacoche’, also known as Mexican corn truffles, has been consumed by populations in Mexico since pre-Colombian times. Since then, its edible popularity has gained wider acceptance, and has expanded to other countries where it is now consumed as an exotic delicacy.

A number of studies have revealed that ‘huitlacoche’ has a high nutritional value, as it is high in protein and in essential amino acids, such as lysine, an amino acid that is commonly deficient in corn protein. Compared to other edible fungi, ‘huitlacoche’ has a higher content of bioactive compounds with antitumoral activities, such as β-glucans, thus serves as a viable functional food candidate [1, 3]. Additionally, *U*. *maydis* produce secondary metabolites having valuable applications, such as glycolipids (cellobiose lipid-ustilagic acid and mannosylerythritol lipids-MEL), siderophores, indole pigments, and protease [4].

*U*. *maydis* is a hemibasidiomycete with a narrow host range, as it only infects maize (*Zea mays*) and its progenitor plant teosinte (*Zea mays* subsp. *parviglumis*). The sexual stage of *U*. *maydis* begins when compatible haploid sporidia mate on young leaf tissues, establishing dikaryotic filaments that invade these tissues, and inducing tumor formation [5]. Within these tumorous tissues, diploid teliospores with thickened cell walls are produced, which in turn can germinate to initiate the next generation cycle [6]. Development of the dikaryon is controlled by the biallelic *a* mating type locus, encoding for the pheromone precursor (*mfa1*/*2*) and the receptor (*pra1*/*2*), that regulates cell fusion [7]. Subsequently, the bE/bW transcription factor, encoded by the multiallelic *b* locus and formed via the interaction of the homeodomain proteins bE and bW, controls the pathogenic stage of this fungus [8]. Thus, only haploid cells with different *a* and *b* loci can mate and produce infectious filaments.

Availability of genomic resources, including a genome sequence, plasmids, selectable markers, constitutive and regulated promoters, critical reporters, along with a relatively short duration to obtain infected plants [9–11] have all contributed to increased knowledge of the biology of *U*. *maydis*, rendering it as a useful and ideal model organism for pursuing cellular and molecular studies. Recently, it has been suggested that *U*. *maydis* in its free life form should be considered for expression of recombinant proteins. In this stage, *U*. *maydis* grows as haploid yeast-like cells that are easily manipulated, thus allowing them to grow in a variety of low-cost production systems, even as substrates in biological waste products [12].

Recombinant organisms providing edible biomass would serve as attractive systems for both expression and delivery of high-value proteins such as subunit vaccines [13–14]. Production of low-cost vaccines that can be administered orally can have a significant impact on world-wide mass immunization efforts [15–16]. Although filamentous fungi are robust systems for the production of recombinant proteins [17], their use for vaccine production has not yet been fully explored. The B subunit of the cholera toxin (CTB) is a potent immunogen associated with protection against *Vibrio cholera* and enterotoxigenic *Escherichia coli* (ETEC) [18]. In addition, it serves as a mucosal adjuvant, as it is used as a carrier for mucosal delivery of chemically- and genetically- coupled antigens [19]. Immunization through oral or nasal administration of CTB-coupled antigens has demonstrated therapeutic immune modulatory activity, even in models of autoimmune diseases [20]. The immunogenicity of CTB is associated with the correct pentamer formation that binds to the GM1 receptor, present in most nucleated cells [19, 21].

In this study, expression of the B subunit of the cholera toxin (CTB) was achieved in corn smut (‘huitlacoche’), following infection of maize with transgenic *U*. *maydis* strains carrying the *CTB* gene, in an effort to explore the feasibility of using corn smut or ‘huitlacoche’ as a novel host for production and delivery of safe oral subunit vaccines. This will contribute to an innovative production platform for vaccines and for other therapeutic compounds.

## Materials and Methods

### Strains and growth conditions

The wild-type strains of *U*. *maydis*, FB1 (*a1b1*) and FB2 (*a2b2*), and the FB2-derivative strains were grown routinely in either liquid YPD (2% yeast extract, 1% peptone, and 1% glucose) or YEPSL (0.4% yeast extract, 0.4% peptone, and 2% sucrose) medium at 28°C, along with continuous agitation on a rotary shaker at 200 rpm. For cloning of the pMF1h-CTB plasmid, *E*. *coli* Top10 strain was used.

### DNA and RNA isolation

Total DNA and RNA from *U*. *maydis* strains and from corn smut galls (teliospores) were isolated as previously described [22–23], respectively.

### Plasmid construction, transformation, and analysis

In order to generate a P*o2tef*::*CTB* fusion, the open reading frame (ORF) of the cholera toxin B subunit (CTB) was amplified using the vector pUC57-CTB as template, carrying a synthetic *CTB* gene optimized for expression in eukaryotic cells. The following primers were used: CTBfwd 5´-TGA*GGATCC*ATGATCAAGCTTAAGTTCGGAGTTTTC-3´(sense) and CTBrev 5´-*TAGGCGCGCCTC*ATCCAGAGGAGTTACCGGAA-3´(antisense), wherein the *Bam*HI and the *Asc*I restriction sites were included, respectively. The constitutive promoter P*o2tef* was amplified from the pMF2-3c plasmid [9] using the primers pOTEFfwd 5´-TG*GGTACC*AACTAGTGGATCCCCCGTAC-3´(sense) and pOTEFrev5´-AGTCA*GAATTC*CCACTCAGGCCTATTATGCC-3´(antisense) with the *Kpn*I and *Eco*RI restriction sites, respectively. Subsequently, amplicons were digested, and then ligated. Using primers pOTEFfwd and CTBrev, the ligation product was diluted 1:10, and used as a template to obtain the P*o2tef*::*CTB* PCR product. The PCR product was digested with *Kpn*I and *Asc*I restriction enzymes, cloned into corresponding restriction sites of the pMF5-1h plasmid, thus replacing the *egfp* coding sequence, and fusing the digested amplicon upstream of the nos terminator.

**Fig 1:**
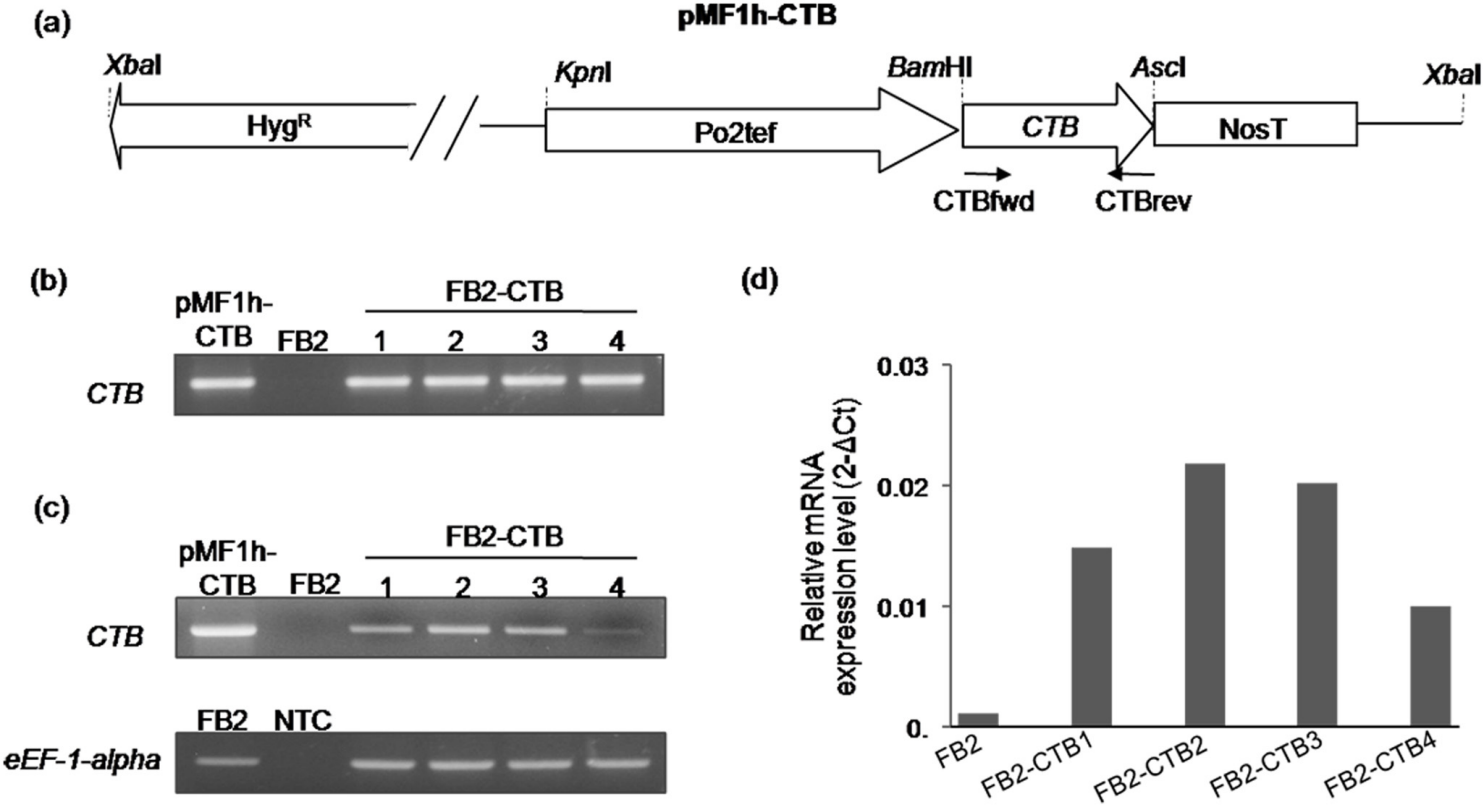
Expression of the *Vibrio cholerae CTB* gene in the yeast-like *U*. *maydis* FB2 strain. (a) Schematic diagram of the pMF1h-CTB plasmid used for transformation of *U*. *maydis* FB2. The *CTB* gene was fused to the constitutive *Po2tef* promoter and to the nopaline synthase terminator (NosT). (b) Specific amplification of the *CTB* gene from the genomic DNA of four FB2-CTB transformants, using the CTBfwd and CTBrev primers. (c) and (d) Expression of the *CTB* gene in four FB2-CTB transformants determined by semi-quantitative RT-PCR and quantitative RT-PCR expression, respectively. Quantitation of the *CTB* gene expressed as relative mRNA expression levels (2^-ΔCt^), was calculated after normalization to the *U*. *maydis eEF-1-alpha* gene, used as control. Total RNA was isolated from WT and *CTB*-expressing FB2 yeast-like cells grown in YPD medium in agitation at 28°C, during 12 h. Genomic DNA from *U*. *maydis* FB2 strain, the plasmid pMF1h-CTB, and a sample without template (NTC), were used as control.

Following sequencing, the resulting plasmid, designated as pMF1h-CTB (Fig 1a), was linearized with an *Xba*I restriction enzyme, and then used to transform *U*. *maydis* FB2 protoplasts as previously described [9]. Cells were grown in YEPSL medium at an OD_600_ of 1.0. After harvest, cells were washed once with SCS [20 mM sodium citrate (pH 5.8), 1 M sorbitol], and resuspended in 2 ml of SCS containing 10 mg/ml of the lytic enzyme of *Trichoderma harzianum* (Sigma-Aldrich). The mix was incubated for 30 min at room temperature for fungal cell wall digestion. The reaction was stopped by adding an SCS buffer, and then washed twice with SCS. Finally, cells were washed once with STC [10 mM TRIS-HCl (pH 7.5), 100 mM CaCl_2_, and 1 M sorbitol], and cells were resuspended in STC.

After adding 5 μL of linearized DNA (1μg/μL) and 1.5 μL of heparin 1(1.0 mg/mL), 50 μL of protoplasts were incubated for 10 min in ice. Then, 500 μL of STC/PEG (10 g of PEG4000 in 15 ml of STC) was added, and the mixture was incubated for 15 min on ice. Briefly, cells were spread in 10 mL of regeneration agar (YEPSL with 1.5% agar, 1 M sorbitol and 400 μg/mL hygromycin B), and overlaid with 10 mL of regeneration agar without antibiotic.

Integration of the plasmid was confirmed by PCR amplification of the *CTB* gene using the CTBfwd and CTBrev primers, and genomic DNA from each of the *U*. *maydis* strains was used as template. The following four transgenic lines were identified, and designated as FB2-CTB 1, 2, 3, and 4 (*a2b2* P*o2tef*::*CTB*).

### *CTB* gene expression

Total RNA was isolated from cells of four transgenic strains of *U*. *maydis* grown for 12 h in YPD medium and from corn smut galls harvested 18 days post-infection (dpi). Following treatment with DNase I (Promega), first-strand cDNA synthesis was performed in a total volume of 20 μL using 2 μg of total RNA with the SuperScript II Reverse Transcriptase (Invitrogen, CA, USA) following the manufacturer´s instructions. PCR was carried out in a 25 μL reaction with 1 μL of RT reaction product used as a template and CTBfwd and CTBrev specific primers. PCR conditions were as follows: 94°C for 5 min, 30 cycles of 94°C for 30 s, 57°C for 30 s, 72°C for 40 s, followed by a final extension of 72°C for 5 min. PCR products were electrophoretically separated on 2% agarose gels, and visualized following ethidium bromide staining. A 140 bp RT-PCR product of the elongation factor gene *eEF-1-alpha* (um00924) was used as a control [24]. For quantitative RT-PCR analysis, total RNA extracted from *U*. *maydis* strains or corn smut galls was treated with DNase I (Invitrogen), and the amount was determined before and after DNase treatment using a NanoDrop ND-1000 UV-VIS spectrophotometer (NanoDrop Technologies). cDNA synthesis and quantitative PCR analyses were done in a 10 μL reaction mixture containing 50 ng of total RNA as template, using the Power SYBRGreen RNA-to-CT1-Step Kit (Applied Biosystems) [25]. The primers used were the qCTBFwd 5´-CGCTTATCTTACAGAAGCTAAGG-3´(sense) and the CTBrev (antisense). Quantification was based on a cycle threshold value wherein the expression level of the *CTB* gene was normalized to transcript levels of the *U*. *maydis* elongation factor *eEF-1-alpha* (um00924) [24].

### Mating and maize infection assay

To evaluate the effects of *CTB* gene expression on mating capacities of FB2-CTB transformants, independent compatible strains were grown overnight, and adjusted to an OD_600_ of 1.0 in sterilized water. Crosses of paired strains were made, 1:1 ratio (v/v), and 4 μL of each cross or strain were inoculated in a minimal medium containing 1% charcoal [26]. Culture plates were sealed, and incubated at room temperature for 48 h.

To assess pathogenicity of transgenic *U*. *maydis*, 8 day-old greenhouse-grown seedlings of creole *Zea mays* (cv. Morado Pardo) were infected by injecting fungal crosses into leaf whorls with the following crosses: FB1 x FB2, FB1 x FB2-CTB 3, and FB1 x FB2-CTB 4. Disease symptoms were scored from 8 to 12 dpi.

Maize ear galls were induced by injecting 10 mL of each strain mixture into silk channel of primary ears, 4–6 days after silking. All plants were maintained under greenhouse conditions, and disease symptoms were scored 15 to 20 dpi. Samples of ‘huitlacoche’ were harvested 18–20 dpi, lyophilized, and then pulverized in a miller analytical.

### CTB immunodetection and quantification

Expression of CTB was assessed using Western blot assays. Protein extracts were obtained by resuspending 30 mg of freeze-dried tissue of corn smut galls in 300 μL of the extraction buffer [750 mM Tris-HCl (pH8.0), 15% (w/v) sucrose; 100 mM β-Mercaptoethanol; and 1 mM PMSF [27] following centrifugation at 12,000 rpm for 15 min and at 4°C. The supernatnat was kept, and 30 μl of protein extracts were mixed with the same volume of 1X reducing loading buffer. Samples were denatured at 95°C for 5 min, and SDS-PAGE was performed in 4–12% polyacrylamide gels. The gel was blotted onto a BioTrace PVDF membrane (Pall Corporation), and subjected to blocking in phosphate saline buffer (PBS) along with 0.01% Tween 20 (PBST) plus 5% fat-free milk. Primary labelling was performed overnight using a mouse anti-CTB antiserum (1:200 dilution). A horseradish peroxidase-conjugated secondary anti-mouse antibody (1:2,000 dilution, Sigma, St. Louis, MO, USA) was added, and the membrane was incubated for 2 h at room temperature. Antibody detection was performed by adding the SuperSignal West Dura solution following the manufacturer’s instructions (Thermo, MA, USA), and signals were detected on an X-ray film following standard procedures. Distinct amounts of pure CTB were included as positive controls (100, 250 and 500 ng per well; Sigma, St. Louis, MO, USA).

### GM1 binding assay

To quantify the amount of protein present in freeze-dried ‘huitlacoche’ biomass, protein extractions were performed using 30 mg of dry biomass resuspended in 300 μl of protein-extraction buffer [50 mM Tris-base (pH 8.0), 40 mM NaCl, 0.5% Triton X-100 (v/v), and 1 mM PMSF]. After vortexing, samples were centrifuged at 14,000 rpm for 15 min at 4°C. Total soluble protein was quantified using the Lowry method using BSA as a standard [28]. CTB content in the soluble protein fraction was determined using the GM1 ganglioside dependent ELISA assay [29]. Briefly, plates were coated with Type III GM1 ganglioside (1.5 μg per well, SIGMA) for a period of 1 h. After washing with PBST, plates were blocked for 1 h at room temperature with 5% fat-free milk, dissolved in PBST. Protein extracts were added, and plates were incubated overnight at 4°C. Primary labelling was performed overnight with a mouse anti-CTB antiserum (1:200 dilution). Horseradish peroxidase-conjugated secondary anti-mouse antibody (1:2000 dilution, SIGMA) was applied for 2 h at room temperature. Color reaction was detected by adding an ABTS substrate (SIGMA). Optical density values were measured at OD405nm using a Multiskan Ascent microplate reader (Thermo Electron Corporation, Waltham, MA, USA) and used to quantify CTB yields as percentages of the total soluble protein (% TSP), using a standard curve prepared with known amounts of the pure CTB protein (0.02–0.2 ng/μl; Sigma).

### Immunogenicity assay

This immunogenicity assay was carried out in accordance with the recommendations listed in the Guide for Care and Use of Laboratory Animals of the National Institute of Health. The protocol was approved by the Committee on Research Ethics of the Chemistry College/University of San Luis Potosí (Permit Number: CEID-2013-004). All efforts were made to minimize animal suffering.

Two groups (n = 4) of 12 week-old female BALB/c mice were randomly established, and subjected to oral administration with one of the following two treatments, 50 mg of freeze-dried FB2-CTB 3 galls (containing 65 μg of oligomeric CTB) or 50 mg of freeze-dried WT galls. Doses were prepared by resuspending the gall powder in 100 μl PBS, and administered on days 0, 7, and 14. Mice were bled at day 21, and sera were collected to determine antibody levels using ELISA analysis [30]. Ninety six-well polystyrene plates were coated overnight with CTB at 0.25 μg/well at 4°C. After blocking with 5% fat-free milk for 2 h, plates were further incubated overnight at 4°C with serial dilutions of mice sera in PBS (1:10 to 1:80 dilutions). Horseradish peroxidase-conjugated secondary anti-mouse antibody (1:2000 dilution, Sigma, St Louis, MO, USA) was applied for 2 h at room temperature, and after washing, signals were detected following incubation with an ABTS substrate for 15 min (SIGMA). Specific antibody levels were expressed as corresponding optical density values measured at OD_405nm_ using a Microplate reader (Thermo Electron Corporation). IgG titers were then calculated as follows: the reciprocal of the higher serum dilution with an OD value above the mean OD value of the WT group plus 2 times its standard deviation.

The Cholera toxin (CT) challenge was performed on day 28 following the procedure previously described [31], but with some modifications [30]. In addition to the two experimental groups treated with either WT or transgenic ‘huitlacoche’, two control mice groups were included. One control consisted of an unchallenged mice group, while a second control consisted of a naïve mice group treated with PBS and challenged with CT. Briefly, either 10 μg of CT diluted in 10% NaHCO_3_ or the vehicle alone were orally administered to food-deprived mice, wherein food has been withheld for a period of 16 h. After a period of 6 h, with access only to water, mice were sacrificed by cervical dislocation, and the entire small intestine, from the pyloric valve to the ileal-cecal junction, was dissected, and weighed. The volume of fluid accumulation (FA) in the small intestine, following challenge, was calculated as described [31], and according to the following: FA = (G/B-G) x 1000; wherein *B* is total body weight (in grams) and *G* is total gut weight (including fluids) (in grams).

### Statistical analysis

ELISA data presented correspond to the geometric means of values obtained per group, while error bars represent standard deviations. Significant differences in antibody levels and fluid accumulation values between pairs of groups are assessed using one-way analysis of variance followed by mean comparisons using Tukey’s test with P values < 0.05 deemed significant.

## Results

### Developing *U*. *maydis* strains over-expressing the *CTB* gene

In order to express the B subunit of the cholera toxin of *V*. *cholerae*, several transgenic *U*. *maydis* strains overexpressing the *CTB* gene, which has been optimized for expression in eukaryotic cells, under the control of the constitutive and strong P*o2tef* promoter were generated (Fig 1a). This promoter is a chimeric fusion of seven tetracycline responsive elements to the basal promoter of the translation elongation factor 2 from *U*. *maydis* [32]. The pMF1h-CTB plasmid was introduced into *U*. *maydis* FB2 strain, and four hygromycin-resistant transformants, designated as FB2-CTB 1, 2, 3, and 4, were found to carry the *CTB* transgene following PCR amplification of the full-length ORF, while no amplification was detected in the FB2 WT (Fig 1b). Moreover, expression of *CTB* in transformed strains was confirmed by semi-quantitative and quantitative RT-PCR (Fig 1c and 1d).

### Production of ‘huitlacoche’ expressing the *CTB* gene

**Fig 2:**
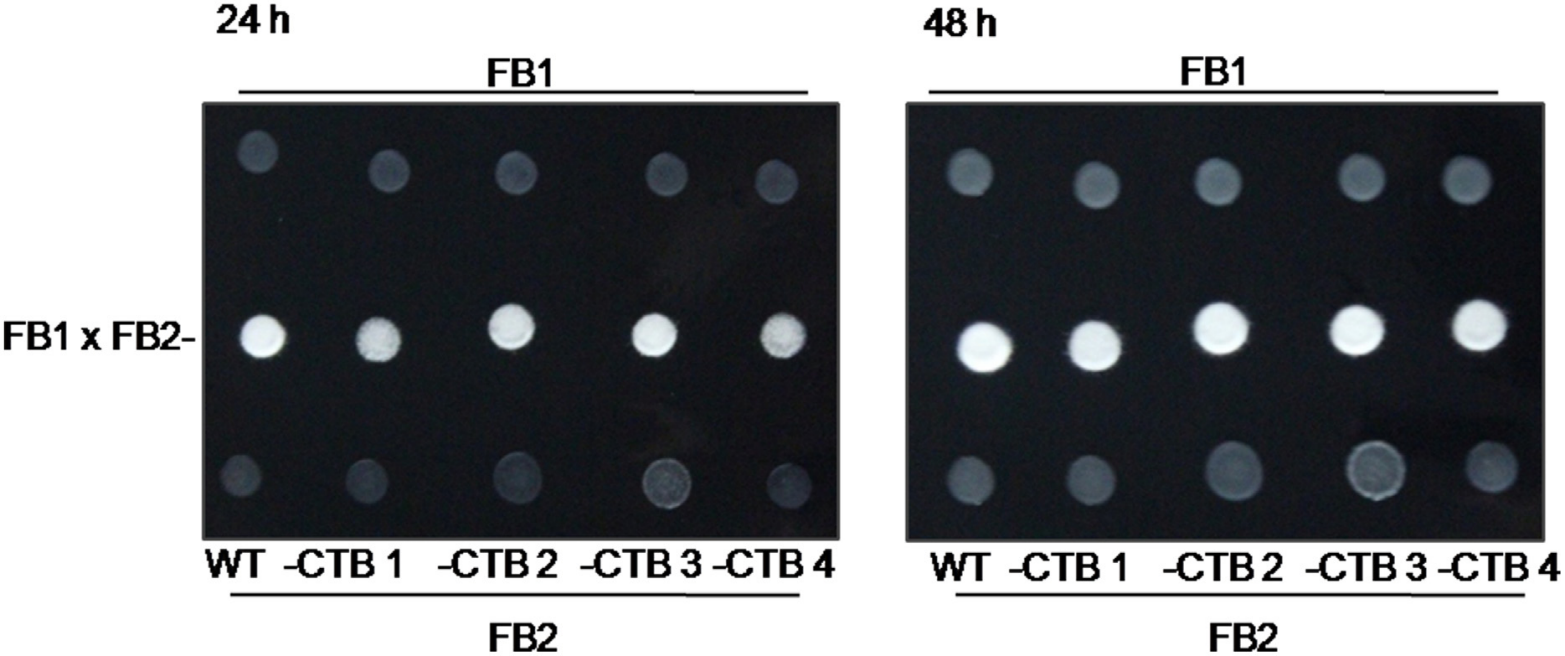
*U*. *maydis* FB2-CTB strains develop fuzzy colonies when are paired withthe FB1 strain. FB2-CTB transformants were co-spotted with their mating partner FB1 strain on a charcoal-containing MM plates. The FB2 x FB1 cross was tested as control. Photos were taken 24 and 48 h after incubation at room temperature.

Prior to inoculation of maize with transgenic *U*. *maydis* FB2-CTB strains, mating capacities of these strains were evaluated. This is a prerequisite for dikaryon establishment, and for pathogenic development. Each of the four transgenic strains, FB2-CTB 1, 2, 3, and 4, were co-spotted with the mating FB1 partner on charcoal-MM plates, and then incubated at room temperature for 48 h. All transgenic FB2-CTB strains developed fuzzy phenotypes, along with white colonies, that were indistinguishable from those of compatible WT strains (Fig 2). This finding suggested that FB2-CTB 1, 2, 3, and 4 strains were competent to mate with the FB1 WT strain.

**Fig 3:**
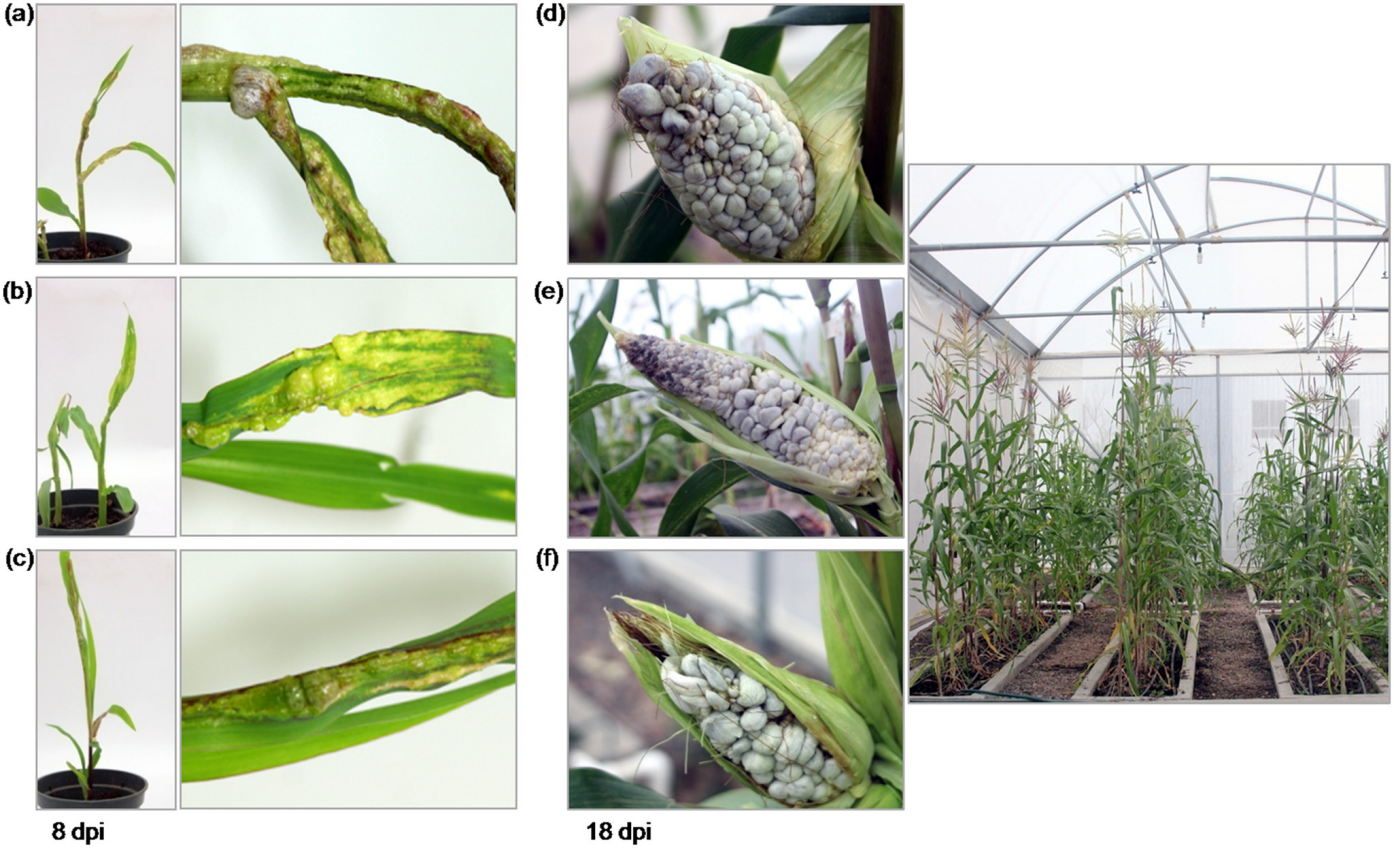
Tumors induced on maize plants, after inoculation with FB2-CTB strains paired with FB1 strain. Leaf tumors induced in 8-day-old maize seedlings inoculated with (a) FB1 x FB2, (b) FB1 x FB2-CTB 3 and (c) FB1 x FB2-CTB 4 crosses. Photos were taken 8 days post-inoculation (dpi). Corn smut galls (‘huitlacoche’) produced by inoculating with (d) FB1 x FB2, (e) FB1 x FB2-CTB 3 and (f) FB1 x FB2-CTB 4 crosses. Plants were grown under greenhouse conditions and photos were taken 18–20 dpi.

**Fig 4:**
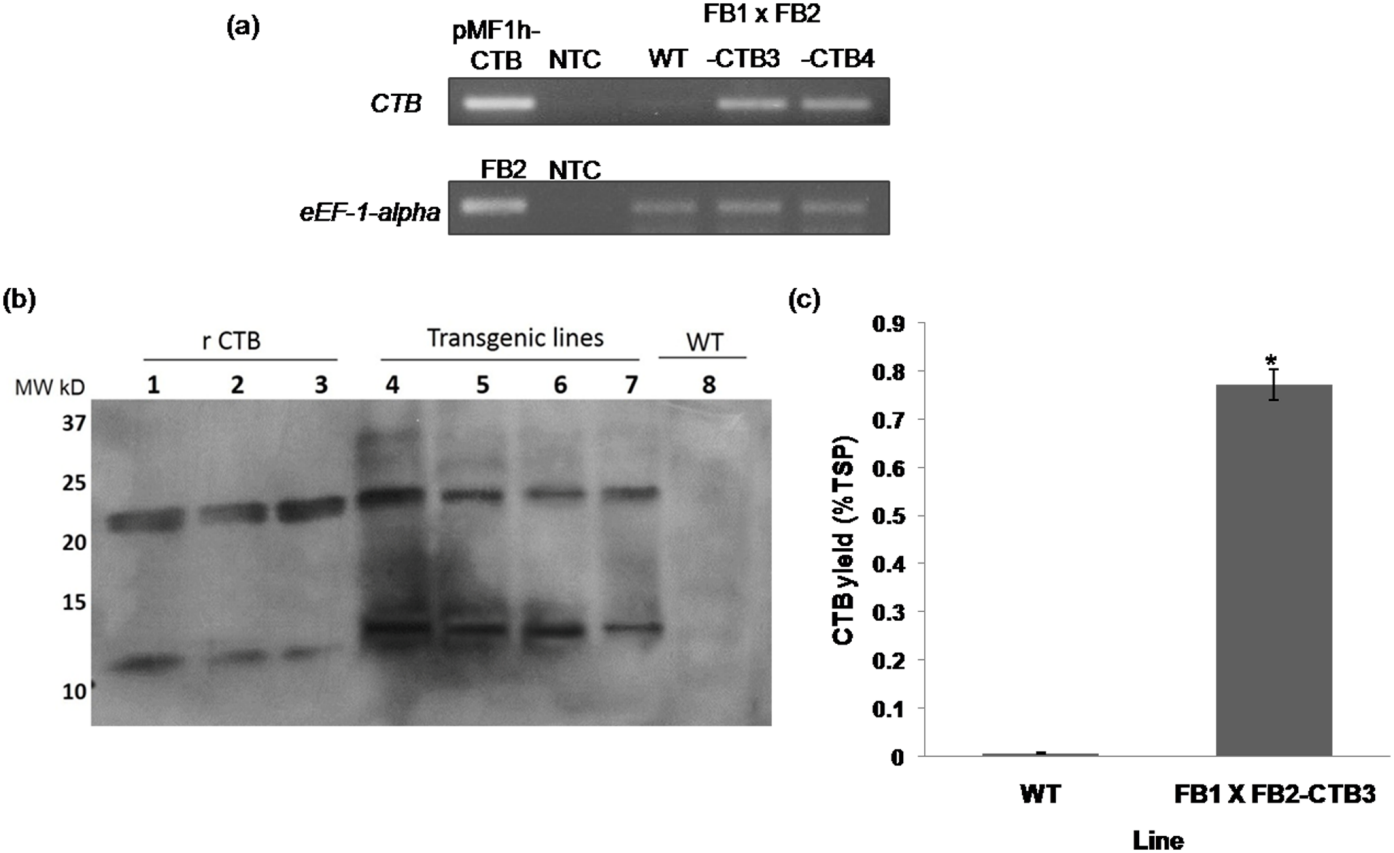
*V*. *Cholerae CTB* gene is successfully expressed in corn smut galls (‘huitlacoche’). Corn cobs were inoculated with the crosses FB1 x FB2-CTB 3 and FB1 x FB2-CTB 4, and FB1 x FB2 as control. (a) Expression analysis of the *CTB* gen in ‘huitlacoche’by semi-quantitative RT-PCR. Total RNA was isolated from fresh WT or transgenic corn smut galls (teliospores). Genomic DNA from *U*. *maydis* FB2 strain, the plasmid pMF1h-CTB, and a sample without template (NTC), were used as control. (b) Detection of the CTB protein in corn smut galls protein extracts by Western Blot analysis. Protein extracts were subjected to SDS PAGE under reducing conditions, blotted, and then labelled with an anti-CTB serum. Lanes 1 to 3, pure CTB standard (500, 250, and 100 ng, respectively); 4 and 5, extracts from ‘huitlacoche’ produced with the FB1 x FB2-CTB3 cross; 6 and 7, ‘huitlacoche’ produced with the FB1 x FB2-CTB 4 cross; and 8, extract from WT ‘huitlacoche’ (FB1 x FB2). (c) Quantification of CTB levels in terms of %TSP by GM1-ELISA assay. Oligomeric CTB was detected in total soluble protein extracts from either FB1 x FB2-CTB 3 galls or WT galls followed by immunodetection with an anti-CTB serum. The Asterisk denotes statistical differences at P< 0.05 versus WT galls.

To assess the pathogenicities of the strains, 8-day-old maize seedlings were inoculated with mixtures of *U*. *maydis* FB1 x FB2-CTB 3 and FB1 x FB2- CTB 4 transgenic strains, and with the control FB1 x FB2 strains. It was found that FB1 x FB2-CTB 3 and FB2-CTB 4 mixtures induced tumor development after 8 dpi, which was similar to that observed for the FBI x FB2 mixture (Fig 3a–3c). Furthermore, transgenic FB2-CTB 3 and FB2-CTB 4 paired with FB1 induced tumors on inoculated ear corns, and these tumors were indistinguishable from those generated following inoculation with the FB1 x FB2 cross (Fig 3d–3f and S1 Table). However, in contrast to WT ‘huitlacoche’, transgenic corn smut galls expressed the *CTB* gene (Fig 4a).

### Immunodetection and quantification of the oligomeric recombinant CTB protein of ‘huitlacoche’

To assess the presence and integrity of the CTB protein in transgenic ‘huitlacoche’, Western Blot analysis was conducted using anti-CTB serum. Extracts of ‘huitlacoche’ infected with mixtures of FB1 x FB2-CTB 3 and 4 showed strong signals of 12 kDa in molecular weight, 3 kDa lower than that of the predicted molecular weight of the CTB protein expressed in *U*. *maydis* (15 kDa). As expected, wild-type corn galls did not show any signals. In spite of the reducing conditions and boiling temperatures of the assay, a 24 kDa band was also detected, likely corresponding to dimers of the CTB protein (Fig 4b). Moreover, a quantitative GM1-ELISA assay revealed that levels of the oligomeric CTB reached up to 1.3 mg g-1 dry weight of gall tissues, corresponding to 0.8% TSP. This finding also demonstrated the proper assembly of the oligomeric structure of CTB, which is critical for its immunogenic activity as binding to GM1 is dependent on this structure and its biological activity (Fig 4c).

### The corn smut CTB recombinant protein confers protection against CT challenge

**Fig 5:**
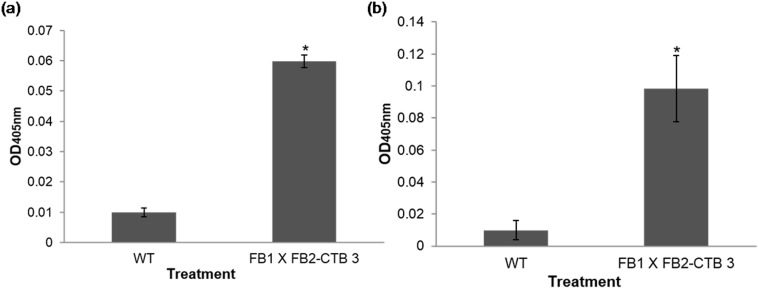
Antibody levels of BALB/c mice immunized with corn smut galls expressing CTB protein. Detection of: (a) anti-CTB IgA antibodies in intestinal washes, and (b) anti-CTB IgG antibodies in sera. Mice immunized with different treatments (biomass from ‘huitlacoche’: FB 1 x FB2-CTB 3 and FB1 x FB2 as control) were bled at day 21, and serum was diluted 1:40. Statistical differences versus the WT group are indicated by an asterisk (*P* < 0.05).

**Fig 6:**
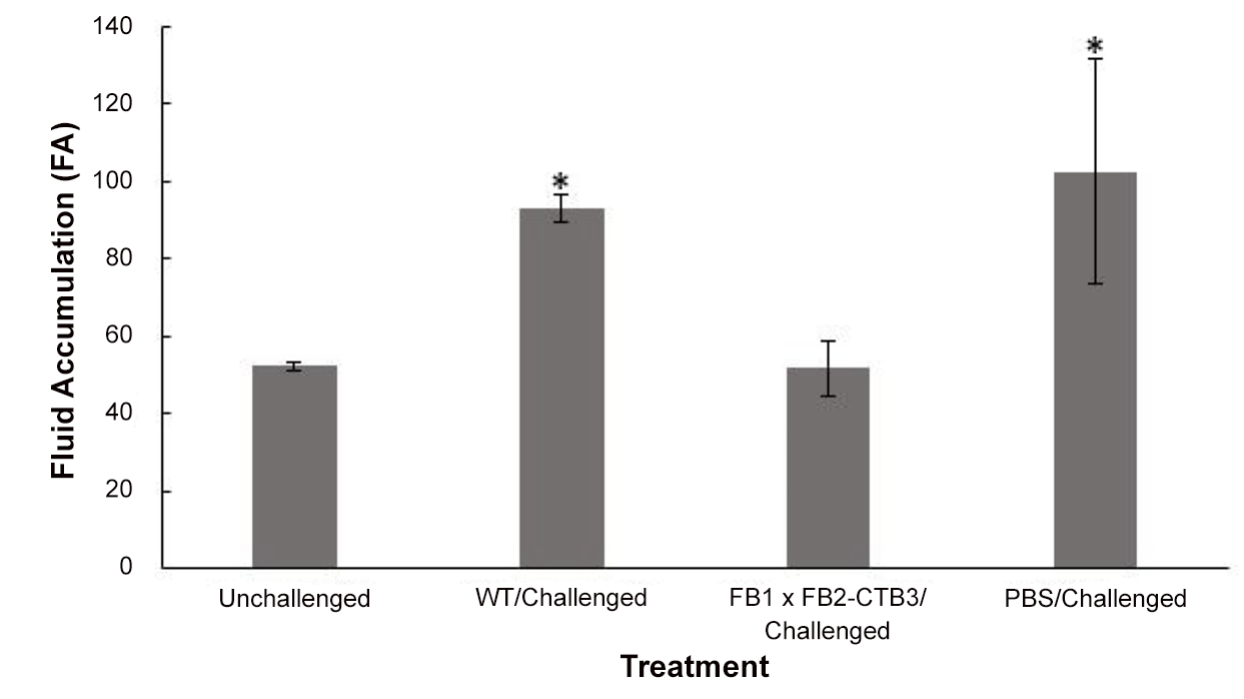
Immunoprotection against CT challenge of BALB/c mice immunized with galls expressing the CTB protein. Mice were immunized with either WT galls or FB1 x FB2-CTB 3 galls, and then challenged with CT. Control groups consisted of an unimmunized mice group challenged with CT (PBS/challenged) and an unimmunized unchallenged mice group (unchallenged). Mice were challenged with 10 μg of CT one week following the last boost. After 6 h of CT toxin administration, mice were sacrificed, and fluid accumulation (FA) was estimated by weighing carcass and small intestines. Comparisons between challenged and unchallenged groups have been made, and significant differences found against the unchallenged group are denoted using an asterisk (*P*< 0.05). Note the lower FA values in the group immunized with FB1 x FB2-CTB 3 galls.

Following a three-week immunization scheme, mice orally immunized with transgenic FB1 x FB2-CTB 3 ‘huitlacoche’showed significantly higher intestinal IgA and serum IgG levels than those immunized with WT ‘huitlacoche’ (Fig 5a and 5b). The anti-CTB IgG titer for the FB1 x FB2-CTB 3 ‘huitlacoche’-immunized group was 40. When mice were challenged with the cholera toxin (CT), fluid accumulation (FA) levels were significantly lower (mean of 51.8) for mice immunized with transgenic ‘huitlacoche’ than those immunized with wild-type tissues (mean of 93.1). In contrast, FA levels of unchallenged mice showed a mean of 52.2 (Fig 6). The observed protective effects induced by the CTB protein produced in transgenic ‘huitlacoche’suggested elicitation of a humoral response in intestinal mucosal tissues in mice.

## Discussion

The CTB protein, as well as various other antigenic proteins, has been expressed in a number of organisms/hosts, including bacteria, yeast, plants (tobacco, tomato, lettuce, and maize), and green algae, *Chlamydomonas reinhardtii* [16, 33–35]. In some systems, either a lack of proper assembly or undesirable addition of a glycan moiety has been reported [35], [36]. Therefore, identifying an alternative platform for production and expression of CTB along with other antigenic proteins that encounter similar problems of expression in host systems is highly desirable.

To develop a low-cost subunit vaccine production platform, we have exploited corn smut galls or ‘huitlacoche’ for production and delivery of a therapeutic recombinant protein as a novel and unique strategy. The CTB protein was produced in corn smut galls by infecting corn ears with a mixture of compatible haploid cells of *U*. *maydis* wherein the mating partner FB2 alone is carrying the *CTB* gene. This has been designed as a biosafety measure, although *U*. *maydis* only infects maize and not humans. Moreover, ‘huitlacoche’ expressing CTB was produced under greenhouse conditions to prevent gene flow and control the release of the transgene, and to optimize the environmental plant growth conditions, including irrigation, as drought is one of the most important limiting conditions for maize production [37]. Developing healthy plants contribute to high amounts of biomass production and protein accumulation, both critical for large-scale molecular farming of therapeutic proteins [38].

Herein, cloning and expression of the *CTB* gene in yeast-like *U*. *maydis* FB2 strain, and subsequent production of the proper structure of the CTB recombinant protein in ‘huitlacoche’ has been successfully achieved. The ‘huitlacoche’ produced after inoculation with different transgenic strains has been identified to express varying levels of the oligomeric CTB protein, reaching yields of up to 1.3 mg g-1 dry weight of galls (0.8% TSP). This demonstrates the high biosynthetic capacity of *U*. *maydis* as many other fungal species [39]. In the molecular farming arena, recombinant protein expression in seeds and viral expression systems are the most promising platforms in terms of yields. Although viral expression vectors have allowed for recovery of very high yields of CTB (up to >1 g kg^-1^ fresh weight) in leaves of *Nicotiana benthamiana*, this expression platform requires purification of the recombinant protein as it is not an appropriate vehicle for oral delivery, due to the presence of toxic compounds [35]. Several efforts to produce CTB or chimeric CTB-based proteins in seed crops have been performed, mainly in rice and maize. In rice, highest yields of chimeric CTB of 1.5 μg g^-1^ dry seed have been reported [40], while 50 μg g^-1^ dry seed yields were reported by [41]. In maize, expression of CTB has reached up to 1.56 μg g^-1^ dry weight [42]. The CTB yields obtained in the ‘huitlacoche’ system are very high when compared to those reported in seed crops.

This is the first report on the use of ‘huitlacoche’ (corn smut) for the production of subunit vaccines. Although long-term storage at room temperature is a major advantage of expression of recombinant therapeutic proteins in maize seeds, serious concerns over gene flow due to wide-range spread of pollen and lengthy process of developing stable transgenic maize lines render this system less desirable [38, 43]. The ‘huitlacoche’ system offers a suitable model to overcome these drawbacks and concerns as it is produced after infecting corn with mixtures of compatible *U*. *maydis* haploid sporidia that are easily transformed. Additionally, the production of purified recombinant proteins from yeast-like *U*. *maydis* grown in batch cultures is also feasible.

Interestingly, the molecular weight of the recombinant protein (12 kDa) is found to be lower than predicted (15 kDa), suggesting that the peptide leader sequence of CTB, first 21 amino acids, has been removed. Previously, it has been suggested that the peptide leader sequence of the CTB protein is involved in translocation of the protein to the endoplasmic reticulum (ER) of eukaryotic systems, wherein proper assembly of the recombinant protein takes place in spite of its glycosylation [35, 36, 44]. Additionally, the GM1 binding assay of CTB produced in ‘huitlacoche’ indicates that the pentameric structure of CTB has been properly assembled in this tissue. As glycosylation is a relevant post-translational modification, this influences immunogenicity, function, and half-life of recombinant protein, and serves as a major challenge in heterologous expression systems. First, undesired glycosylation of antibodies and hormones may become immunogenic leading to undesired immune responses resulting in tissues damage [45]. Second, glycosylation patterns in target antigens might enhance immunogenicity of the candidate vaccine, and accounting for vaccine efficacy [46]. Previously, it has been reported that *U*. *maydis* has an unconventional secretory system that prohibits transfer of proteins through ER [47]. Thus, this serves as a convenient system for expression of proteins that do not require this modification. However, expression of glycosylated proteins is also suitable as the glycosylation pathway of *U*. *maydis* seems to be more similar to that of mammals than that of yeasts as *Saccharomyces cerevisiae* [48].

More importantly, oral immunization of BALB/c mice with transgenic ‘huitlacoche’ has revealed that the ‘huitlacoche’-produced recombinant CTB protein elicits a high level of immunogenicity in test mice. Although immunogenicity of recombinant CTB protein in other host systems has been reported, the high level of immunogenicity observed in ‘huitlacoche’is highly desirable. Furthermore, the protective potential of the elicited immune response, determined in CT-challenged immunized mice and measured by FA values, has demonstrated the neutralizing activity of the recombinant CTB protein in ‘huitlacoche’. In addition, assessment of the immune protection in *V*. *cholera* or ETEC infection models will provide valuable and critical information for use as a candidate vaccine for prevention of these diarrheal diseases that are highly prevalent in developing countries [49]. As CTB is also recognized as a tolerogenic immunogen associated with the expansion of regulatory T cells, the use of this expression system for developing subunit vaccines against autoimmune diseases will contribute to new research thrusts [50]. Future studies should pursue determination of the effects of distinct formulations of this recombinant CTB on immunogenicity. For example, the effects of particle size or the use of gelatin pills to ensure proper dosage are pertinent research objectives. In addition, studying the effect of thermic treatments, e.g. pasteurization, on the vaccine shelf life would be relevant.

To our knowledge, filamentous fungi have not been extensively explored as a platform for the production of recombinant proteins, thus, this study provides a new model of edible fungal species that can be used as a biofactory for production of therapeutic proteins, such as subunit vaccines. The ‘huitlacoche’ biomass also serves as an economic and safe production system for an oral subunit vaccine and at low-cost, as purification of the recombinant protein is not necessary.

## Supporting Information

S1 Table Yield of ‘huitlacoche’ produced after inoculation of corn cobs with the *U*. *maydis* F1 x FB2-CTB 3 and the FB1 x FB2-CTB 4 mixtures. (DOCX)
