# Supplementary material for: The Corn Smut (‘Huitlacoche’) as a New Platform for Oral Vaccines
Source: PLoS One. 2015 Jul 24;10(7):e0133535. doi: 10.1371/journal.pone.0133535 (PMC4514630; doi:10.1371/journal.pone.0133535)
Supplement: S1 Table — (DOCX) [file pone.0133535.s001.docx]

**Table S1** Yield of ‘huitlacoche’ produced after inoculation of corn cobs with the *U. maydis* F1 x FB2-CTB 3 and the FB1 x FB2-CTB 4 mixtures.

Corn smut galls were harvested between 18 to 20 dpi. WT corn smut galls (FB1 x FB2) were produced as control

| **Cross** | **Inoculated**  **cobs** | **Harvested ‘huitlacoche’** | **Infection**  **(%)^a^** | **Total weight**  **(g)^b^** | **Yield**  **(%)^c^** |
| --- | --- | --- | --- | --- | --- |
| FB1 x FB2 | 53 | 48 | 90 | 6862.2 | 100 |
| FB1 x FB2-CTB 3 | 53 | 50 | 94 | 6261.2 | 91 |
| FB1 x FB2-CTB 4 | 53 | 43 | 81 | 6180.1 | 90 |

^a^. Expressed as percentage of the total number (n) of infected cobs

^b^. Total weight from the total number (n) of harvested ‘huitlacoche’

^c^. Expressed as as percentage of the total ‘huitlacoche’ weight of the FB1 x FB2 cross, which was assigned as 100%
